# Octarellin VI: Using Rosetta to Design a Putative Artificial (β/α)_8_ Protein

**DOI:** 10.1371/journal.pone.0071858

**Published:** 2013-08-19

**Authors:** Maximiliano Figueroa, Nicolas Oliveira, Annabelle Lejeune, Kristian W. Kaufmann, Brent M. Dorr, André Matagne, Joseph A. Martial, Jens Meiler, Cécile Van de Weerdt

**Affiliations:** 1 GIGA-Research, Molecular Biology and Genetic Engineering Unit, University of Liège, Liège, Belgium; 2 Departments of Chemistry and Pharmacology, Center for Structural Biology, Vanderbilt University, Nashville, Tennessee, United States of America; 3 Laboratoire d’Enzymologie et Repliement des Protéines, Centre for Protein Engineering, University of Liège, Liège, Belgium; UMR-S665, INSERM, Université Paris Diderot, INTS, France

## Abstract

The computational protein design protocol Rosetta has been applied successfully to a wide variety of protein engineering problems. Here the aim was to test its ability to design *de novo* a protein adopting the TIM-barrel fold, whose formation requires about twice as many residues as in the largest proteins successfully designed *de novo* to date. The designed protein, Octarellin VI, contains 216 residues. Its amino acid composition is similar to that of natural TIM-barrel proteins. When produced and purified, it showed a far-UV circular dichroism spectrum characteristic of folded proteins, with α-helical and β-sheet secondary structure. Its stable tertiary structure was confirmed by both tryptophan fluorescence and circular dichroism in the near UV. It proved heat stable up to 70°C. Dynamic light scattering experiments revealed a unique population of particles averaging 4 nm in diameter, in good agreement with our model. Although these data suggest the successful creation of an artificial α/β protein of more than 200 amino acids, Octarellin VI shows an apparent noncooperative chemical unfolding and low solubility.

## Introduction

### The Inverse Protein-folding Problem

The aim of *de novo* protein design, often called the “inverse protein-folding problem”, is to find amino acid sequences compatible with a given protein tertiary structure. The primary structure of a protein largely determines its tertiary structure [1], [2], and the number of protein sequences compatible with a given fold is limited. Solving the inverse protein-folding problem is therefore a stringent test of our understanding of sequence-structure relationships in proteins. Improving this understanding should help to solve the “protein-folding problem” *per se*: predicting what tertiary structure a given amino acid sequence will adopt. This should ultimately enable us to engineer proteins with custom functions and properties.

### Attempts to Model the TIM-barrel Fold

*De novo* construction of a stable, soluble protein of more than two hundred amino acids is a challenge that remains to be met. Reported successes in designing large artificial proteins involved creating new proteins by assembling, in variable number, multiple copies of a same motif of no more than 40 amino acids long. [3], [4]. Attempts to design longer sequences *de novo* have focused on the TIM-barrel fold [5], [6], [7], [8].

The (β/α)_8_ fold, also known as the TIM-barrel fold, is a very widespread protein topology. It is shared by at least 23 superfamilies in the Structural Classification Of Proteins (SCOP) database [9] and is the most common enzyme fold in the Protein Data Bank (PDB) [10]. It is commonly accepted that more than 10% of all enzymes with known structure contain the (β/α)_8_ fold [11], [12]. Though more than 76 different sequence families have been listed, they all share a very well defined topology.

Typically, TIM-barrels have between 200 and 250 residues. They can be schematically represented as an eightfold repetition of (βα) units organized in two circular layers of secondary structures. The inner layer consists of eight parallel β-strands, surrounded by an external layer of eight α-helices. The β-strands are paired by a strong hydrogen bond network and form a completely enclosed parallel barrel. The catalytic activity of such proteins is nearly always located on the βα side of the protein, whereas the αβ loops are believed to play a crucial role in stabilizing the structure [13].

Despite the relative ease with which nature creates these (β/α)_8_ barrels, attempts to design artificial TIM-barrels *de novo* have had limited success. Early work, including efforts leading to some of the previous versions of Octarellin, yielded poorly soluble proteins that were hard to characterize and appeared to form molten globule species [5], [6], [8], [14], [15], [16]. There is one notable exception where computational *de novo* design of an artificial (β/α)_8_ barrel based on an idealized framework yielded a stable protein appearing to adopt a well-defined tertiary structure [7], but the solubility and stability of this protein were low in the long term, making it impossible to characterize its 3D structure by X-ray diffraction.

### Today’s Powerful Computational Design Methods

The design protocols employed in the above-mentioned studies relied heavily on a combination of chemical intuition and bioinformatic data collected from a limited set of natural sequences. Since then, the number of available crystal structures has increased substantially, and powerful computational methods have emerged, enabling the automated design of sequences folding into a desired topology [17].

The new computational methods use a search function that can rapidly sample the conformational and sequence space and an energy function that can identify minimal energy sequence/conformation pairs [18], [19]. The complexity of the conformational search space can be reduced by sampling discrete amino-acid side-chain conformations observed frequently in solved structures [20], [21], [22]. While the backbone of the protein is usually kept fixed, the side-chain conformations are altered by systematic [23] or random [19] substitutions of rotamers. Recent protocols alternate this side-chain conformational search with an all-atom energy minimization [24], [25]. The energy functions used to evaluate the resulting sequences rely on statistical parameters derived from databases of known protein properties [19], [20], [21], [22], [26]. These “knowledge-based potentials” increase the accuracy of scoring functions for evaluating the designed sequences.

### Using Rosetta to Design an Artificial (β/α)_8_ Barrel

Amongst the programs implementing the new approach, Rosetta has been successfully applied to a wide variety of design problems [27]. Highlight achievements include thermo-stabilizing an enzyme [28], creating a new backbone conformation in a beta turn [29], redesigning the specificity at protein-protein interfaces [30], [31], designing novel enzymes based on existing protein scaffolds [32], [33], [34], and designing an entirely new protein topology [17]. This last result was particularly exciting, as the designed protein, Top7, counts 100 amino acids and is soluble, monomeric, and exceptionally stable. These properties have made it possible to determine a high-resolution crystal structure matching the design model to within 1.2 Å. This success has prompted us to try to push the size limit further. We thus present circular dichroism, dynamic light scattering, and intrinsic fluorescence emission data on Octarellin VI, a 216-amino-acid protein designed with Rosetta to adopt the TIM-barrel fold.

## Materials and Methods

### Structure Design

**Figure 1:**
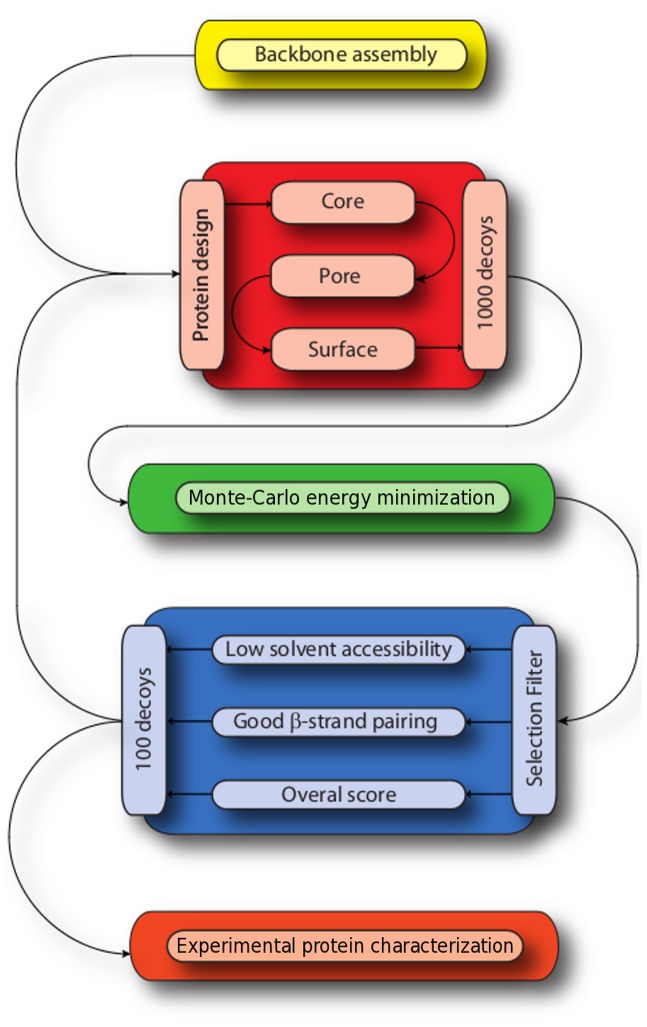
Schematic overview of the design process leading to Octarellin VI. The alternate steps of sequence design, strain removal and filtering procedure are described.

To define a protein with a (β/α)_8_ barrel fold, we worked with the RosettaDesign software. The protocol used by RosettaDesign has been explained and detailed previously [17], [27], [35], and the whole process is summarized in Figure 1. To obtain the desired α/β barrel fold, the objective here was to assemble β-strands (E), α-helices (H), and loops (L) so as to give our backbone an idealized α/β barrel topology: a central sheet of eight parallel strands surrounded by eight helices. A schematic Cα trace consistent with the canonical geometrical features of TIM barrel helix and strand secondary structure was assembled, using as starting point the coordinates of the backbone of our previous design, Octarellin V [7]. For construction of loop regions, six-residue fragments of PDB proteins displaying the secondary structure pattern [E,E/L,L,L,L,L,L/H,H] for βα-loops or [H,H/L,L,L,L,L,L/E,E] for αβ-loops were extracted with the Rosetta loop-building protocol [36]. Those compatible with the geometric coordinates of strands and helices were attached. During the initial design phases, loop positions were set as glycines. A total of 6,000 backbone conformations were constructed with variations in loop conformations.

**Figure 2:**
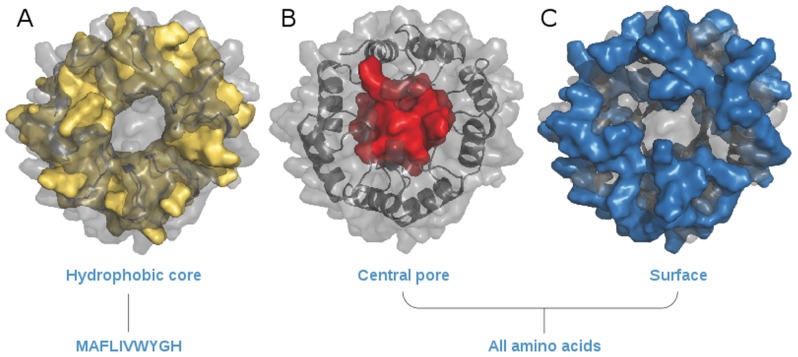
Regions defined by the classification of backbone positions during the hierarchical sequence design procedure. Only amino acids with hydrophobic side chains were used in the core region (A); all residues except cysteine were allowed in the pore (B) and surface (C) regions.

Each backbone position was classified as being either surface, core, or pore (even when it is known not to be a real pore, we keep this nomenclature for historical reasons) by visual inspection. Surface positions are amino acids belonging to α-helices and loops and are largely exposed to the solvent. Positions projecting the side chain into the space between α-helices and β-strands belong to the core. Pore positions are amino acids belonging to the β-strands and whose side chains project towards the inner barrel. As interactions between side chains of different regions are very limited, the three regions were designed sequentially to reduce the computational demand and thus allow the use of larger rotamer sets (Fig. 2). In the protein core, which was designed first, amino acids were restricted to MAFLIVWYGH. In the pore and surface regions, all amino acids except cysteine were allowed. Ten independent design simulations were performed for each of the hundred backbone conformations with the lowest Rosetta energy, generating a total of 1,000 models.

Each of these designed structures was subjected to relaxation with Rosetta’s Monte-Carlo energy-minimization protocol. To select models for the next round of design and refinement, a series of filters were applied. The filter criteria applied were that (i) suitable structures should maintain a tight hydrogen-bonding network in the β-barrel of the protein, as evaluated by the Rosetta backbone hydrogen bonding energy; (ii) side chains should be tightly packed so as to exclude solvent from the core, as evaluated by the Rosetta solvent accessible surface area (SASA) measurement; (iii) accepted structures should have minimal Rosetta full-atom energies. After each energy minimization run, the hundred lowest-energy models meeting these criteria were moved forward for further sequence design/backbone optimization. The iterative design process was terminated after five cycles.

### Model Selection for Experimental Validation

The best structures from the last round were inspected visually and ranked according to (i) the presence of at least one aromatic residue in the protein core (to facilitate experimental studies) and (ii) the extent to which each protein’s amino acid composition, loop geometry, surface hydrophilicity, and predicted secondary structure matched those of natural TIM-barrel proteins.

Further targeted rounds of design were performed to eliminate three hydrophobic patches on the protein surface in the best-ranked design. In these additional design simulations, the residues in the three problematic patches were restricted to ones with small hydrophilic side chains so as to avoid protein aggregation. This phenomenon is not explicitly considered in the Rosetta energy function and was similarly adjusted in previous designs with Rosetta [17]. The final model was called Octarellin VI.

### Analysis of the Final 3D Model with External Softwares

To check the accuracy of our 3D model, we performed stereochemical analysis with a Ramachadran plot [37] and energetic analysis with the Anolea [38], [39] and ProsaII [40] webservers, using in both cases the default parameters.

### Fold Recognition

The sequence of the final model was analyzed with the help of the I-Tasser [41], [42], PsiPred [43], [44], and 3D-Jury [45] webservers, with the default parameters.

### Molecular Dynamics Analysis

To test protein stability and the validity of sequence-structure relationship predictions, a molecular dynamics analysis was performed. Using the software Gromacs [46] and the forcefield OPLS/AA, we first performed an energy minimization by “steepest descent”. We then performed a short, 20-ps molecular dynamics simulation for equilibration with the solvent and then 10 full 5-ns molecular dynamics simulations to test the stability of the designed protein and any changes in it. The entire simulation was done with explicit solvent at 300 K. The values obtained for each trajectory were averaged, the root mean square of the deviation (rmsd) of the backbone being monitored throughout the MD simulation to determine structural convergence. Information about secondary structure, radius of gyration, and the rmsd of the backbone and of each amino acid was extracted from the trajectories.

### Comparison with Natural TIM-barrel Proteins

The final model was also compared with crystallized natural TIM-barrel proteins. Eighteen proteins displaying the (β/α)_8_ fold were selected from the PDB. Each of these structures has a resolution better than 2.2 Å, is known to be a monomer under biological conditions, possesses a chain length of less than 500 residues, and its sequence has less than 70% of identity to that of any other protein in the set. The PDB codes of the eighteen proteins are: 1A53, 1AJ2, 1B54, 1BQC, 1CNV, 1EDG, 1EOK, 1G0C, 1I1W, 1J6O, 1NQ6, 1O1Z, 1PYF, 1UJP, 1VFL, 1WDP, 2CYG, and 7A3H. In addition to energy (Table1) and solvent accessible surface area (SASA) analysis with Rosetta, our synthetic (β/α)_8_ barrel protein was compared with our set of natural TIM-barrel proteins as regards amino acid composition (Table 2) and predicted secondary structure. Agreement in secondary structure prediction (the *SS_score_*) was quantified by comparing the DSSP-assigned secondary structure [47] with the probability assigned to that secondary structure type in the three-state prediction by JUFO [48]. The following equation was used to calculate a score:
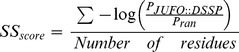
where *P_JUFO::DSSP_* is the probability assigned by JUFO to the DSSP-assigned secondary structure and *P_ran_* = 0.33 is the probability of randomly assigning the correct secondary structure assuming each secondary structure type is equally probable.

**Table 1:** Rosetta energy values.

Rosetta Energetics	Average value for Octarellin VI	Average value for the control set
**Rosetta Energy Units per residue**	−2.43	−2.29±0.18
**Normalized Solvent Accessible Surface Area**	3.80	1.49±1.09
**Solvent Accessible Surface Area Probability**	0.34	0.46±0.05
**Secondary Structure Propensity Energy**	−0.62	−0.55±0.1

**Table 2:** Amino acid composition and properties.

	Value for Octarellin VI	Average value for the control set
Amino acid identity	Percentage (%)	Percentage (%)
Ala	8.8	8.0±2.4
Arg	5.1	4.2±1.5
Asn	7.9	6.1±2.0
Asp	1.9	6.1±1.4
Cys	0.0	1.0±1.0
Gln	7.4	3.4±1.4
Glu	6.0	7.0±3.2
Gly	18.1	7.4±1.7
His	5.1	2.2±1.3
Ile	3.2	6.3±2.1
Leu	8.3	8.4±2.5
Lys	3.7	5.6±2.6
Met	0.5	1.8±1.1
Phe	6.0	3.9±1.0
Pro	0.9	4.3±1.5
Ser	4.2	6.6±1.5
Thr	1.9	4.6±1.4
Trp	5.6	1.7±1.1
Tyr	3.7	3.9±1.5
Val	1.9	7.1±1.8
**Side chain nature**	**Percentage (%)**	**Percentage (%)**
Aliphatic	41.7	43.5±3.7
Aromatic	20.4	11.8±2.4
Small	31.0	21.9±4.6
Long and flexible	22.7	22.0±4.4
Beta-branched	12.0	20.3±3.3
Charged	16.7	22.8±6.2
Negative	7.9	13.0±3.6
Positive	8.8	9.8±3.4
Polar	26.4	23.9±3.8
Polar charged	43.1	46.7±3.3

### Protein Expression and Purification

The gene corresponding to the computationally designed protein Octarellin VI was purchased from BlueHeron Biotechnologies. The gene construct was cloned into the expression plasmid pET-22b (Novagen) and expressed in *E. coli* BL21(DE3) in fusion with a C-terminal hexahistidine tag. Cells transformed with pET22b-Octarellin VI were grown at 37°C in LB containing 100 µg/ml ampicillin. When the culture reached OD_600_ = 0.6, production was induced by addition of isopropyl β-D-1-thiogalactopyranoside at 1 mM final concentration. After 4 h, the cells were harvested by centrifugation. Very good Octarellin VI expression was achieved in *E. coli*, but the protein was found in the insoluble fraction of the bacteria. Inclusion bodies were isolated by resuspending the bacterial pellet in 25 mM Tris-HCl pH 8.5, 500 mM NaCl and rupturing the cells by sonication. After centrifugation of the homogenate, the inclusion-body-containing pellet was washed, first with 25 mM Tris-HCl pH 8.5, 500 mM NaCl and 1% Triton X-100, then three times with the same buffer without Triton. Washed inclusion bodies were solubilized in 25 mM Tris-HCl (pH 8.5), 6 M guanidine chloride. All subsequent purification procedures were performed in this buffer. Denatured protein solution was loaded onto an immobilized metal affinity chromatography (IMAC) matrix charged with the Ni^2+^ ion (IMAC Sepharose HP, XK 16/20 column, GE Healthcare). The protein was eluted with an imidazole gradient (0–500 mM). Fractions containing Octarellin VI were pooled and concentrated before size exclusion chromatography (SEC) on an XK 16/70 Sephacryl S-100 column (GE Healthcare).

### Refolding

Refolding conditions were determined by following the screening procedure described by Vincentelli and co-workers [49]. The best conditions for Octarellin VI refolding were 1∶20 (v/v) dilution in a vigorously stirred solution containing 25 mM Tris-HCl, 500 mM L-arginine, and 100 mM 3-(1-pyridinio)-1-propanesulfonate (NDSB-201) (pH 8.5) followed by incubation at 4°C overnight. Precipitated protein was removed by centrifugation and the refolding solution was concentrated to 1 mg/ml. The concentrated protein solution was dialyzed twice against 10 mM Tris-HCl (pH 8.5). Precipitated protein was again removed by centrifugation and the supernatant filtered with a 0.22-µm filter. Alternatively, when a refolding additive compatible with CD measurements was required, NV10 (Expedeon) was used. In this case, the protein unfolded at 2.6 mg/ml in 6 M urea, 10 mM phosphate buffer, pH 8.0 was refolded by 10-fold dilution in 10 mM phosphate buffer, pH 8.0 containing 1 mg/ml NV10. The refolded protein was extensively dialyzed against 10 mM phosphate buffer, pH 8.0 (to remove urea) and filtered through a 0.45-µm filter. All protein concentrations were determined by measuring absorbance at 280 nm and using the theoretical values of both the extinction coefficient (78,520 M^-1^cm^-1^) and molecular mass (MM = 25,504 Da) calculated with the ExPASy Protparam tool (http://www.expasy.org/tools/protparam.html).

### Dynamic Light Scattering (DLS)

DLS measurements were performed with a Malvern Zetasizer NanoS instrument fitted with a 633-nm laser and a Peltier cell-holder. Data were recorded with a non-invasive backscatter detection angle of 173° at 25°C. A 45-µl “small-volume” 3-mm-path quartz cell containing the protein at 5 µM in 10 mM Tris-HCl (pH 8.5) or 25 mM Tris-HCl, 2 M L-Arginine (pH 8.5) was used. Eleven 10-s runs were performed and averaged. The resulting measurements were collected, analyzed, and correlated with the help of DTS software (Version 5.03) provided by the manufacturer. Solvent viscosity was measured with an AND SV-10 vibro viscometer. Heat-induced protein denaturation was observed under the same conditions. The temperature was increased from 25°C to 95°C by increments of 1°C. Samples were allowed to equilibrate for two minutes before data acquisition.

### Fluorescence Measurements

Fluorescence emission spectra were recorded at 25°C with a Perkin–Elmer LS-50B spectrofluorimeter. The protein concentration was 3 µM in 10 mM Tris-HCl (pH 8.5) and the urea concentration was varied from 0 to 8 M. A stirred cell with a 1-cm pathlength was used. Emission spectra were recorded five times from 300 to 440 nm (excitation at 280 nm) and averaged. The excitation and emission slit-widths were 3.2 nm and the scan rate was 100 nm min^-1^.

### Circular Dichroism Measurements

Circular dichroism (CD) measurements were performed at 20°C with a Jasco J-810 spectropolarimeter equipped with a six-cell Peltier holder, in either the far-UV (190–250 nm) or the near-UV (250–310 nm) region, using a protein concentration of 3 or 39 µM and a cell pathlength of 0.1 or 1 cm, respectively. Spectra were acquired at a scan speed of 10 nm min^-1^, with a 1-nm bandwidth and a 4-s response time. The spectra were measured four times in the presence of NV10 (Expedeon), a synthetic polymer preventing protein aggregation, stabilizing the proteins in solution, and allowing spectroscopic characterization by circular dichroism. The spectra were averaged and corrected by subtraction of the buffer spectrum obtained under identical conditions. Calculation of secondary structures from analysis of the CD data was done with the CONTINLL [50], [51], CDSSTR [52], [53], and SELCON3 [54], [55] algorithms provided by the DichroWeb analysis server [56], [57]. Two protein reference databases (4 and 7) were used and the results obtained with the individual algorithms were averaged; the standard deviations between the calculated secondary structures are reported in Table 3. For thermal and chemical unfolding measurements at a fixed wavelength (222 nm), the compound NV10 was not added.

**Table 3:** Secondary structure determined by CD.

	Helix (%)	Strand (%)	Turn (%)	Unordered(%)
**Octarellin** **VI**	34±3 (45.8)	18±2 (15.3)	19±1 (7.9)	29±2 (31)
***T. maritima*** **TIM**	36±1 (45.6)	18±1 (15.1)	19±1 (11.1)	27±1 (28.2)

Values in parentheses were obtained from the 3D coordinate files with the DSSP software [47].

### Urea- and Heat-induced Unfolding

For urea-induced unfolding, protein samples were incubated overnight at 25°C in the presence of various concentrations of urea ranging from 0 to 8 M in 10 mM Tris-HCl buffer (pH 8.5). The protein concentration was 3 µM. The denaturant concentration was determined from refractive index measurements [58] performed with a R5000 hand refractometer from Atago. For heat-induced unfolding, the same buffer and protein concentration were used. The protein sample was heated by increasing the temperature monotonically from 25°C to 92°C at the rate of 0.5°C/minute. In chemical and heat unfolding experiments, transition curves were obtained by monitoring, respectively, the shift of the maximum fluorescence emission wavelength (λ_max_) and the change in CD signal intensity at 222 nm.

## Results

### Designing an Idealized Artificial TIM-barrel Protein

**Figure 3:**
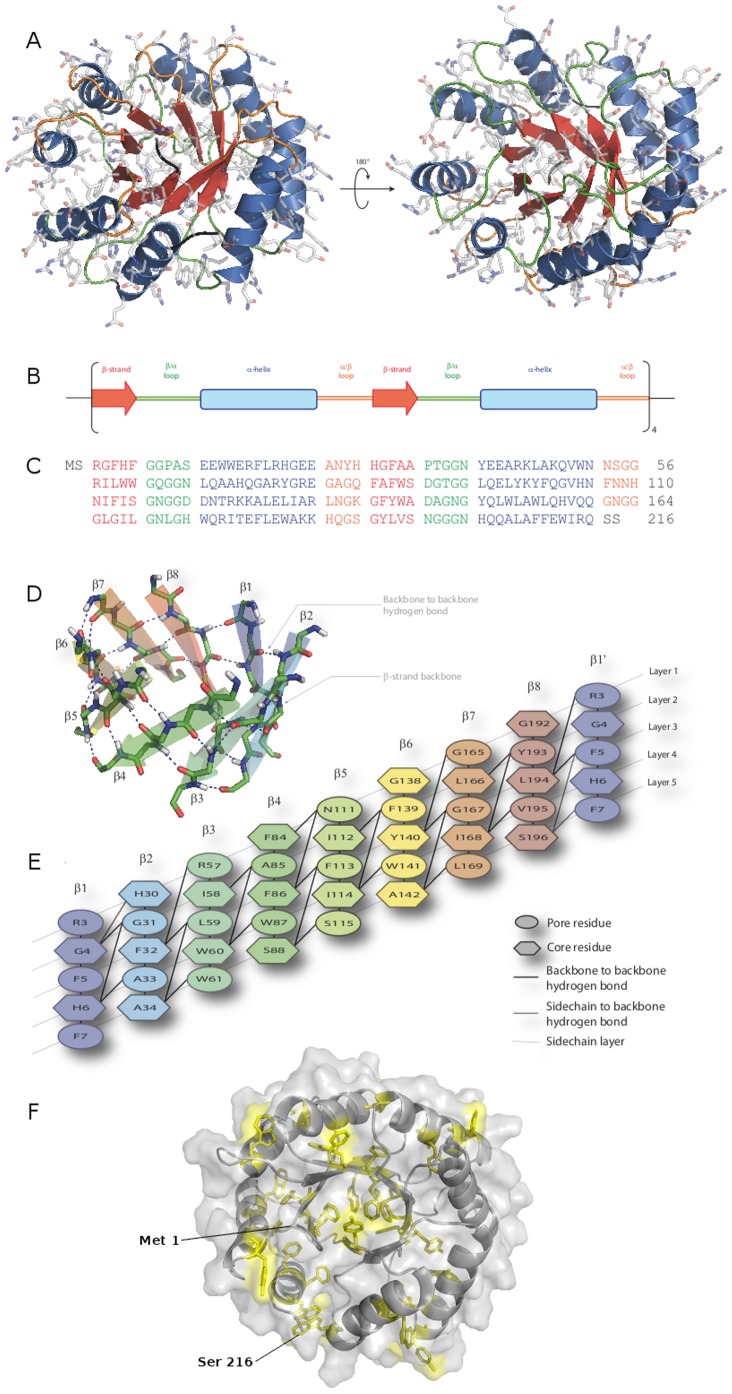
Schematic representation of the Octarellin VI model. The tertiary (A), secondary (B) and primary (C) structures are shown. (D) Schematic overview of the central β-barrel of the artificial protein model, showing the hydrogen bond network. (E) The nearly ideal pattern expected for a TIM-barrel protein. (F) Aromatic amino acids disposition into the tertiary strucure.

An idealized (β/α)_8_ backbone was assembled. Sequence design was alternated with energy minimization steps in an iterative process. Models taken from one cycle to the next were selected by application of a filter (see Methods). Finally, after five iterations, targeted rounds of design were performed to eliminate hydrophobic patches and discourage aggregation. In all, more than 5000 different sequences were tested in the whole design process. The final selected model was named Octarellin VI, because it is the sixth Octarellin created in our laboratory. Figure 3 represents the final 3D model, showing a diagram of the different structural elements present in it.

### The Designed Protein Structure shows Native-like in Silico Characteristics

**Figure 4:**
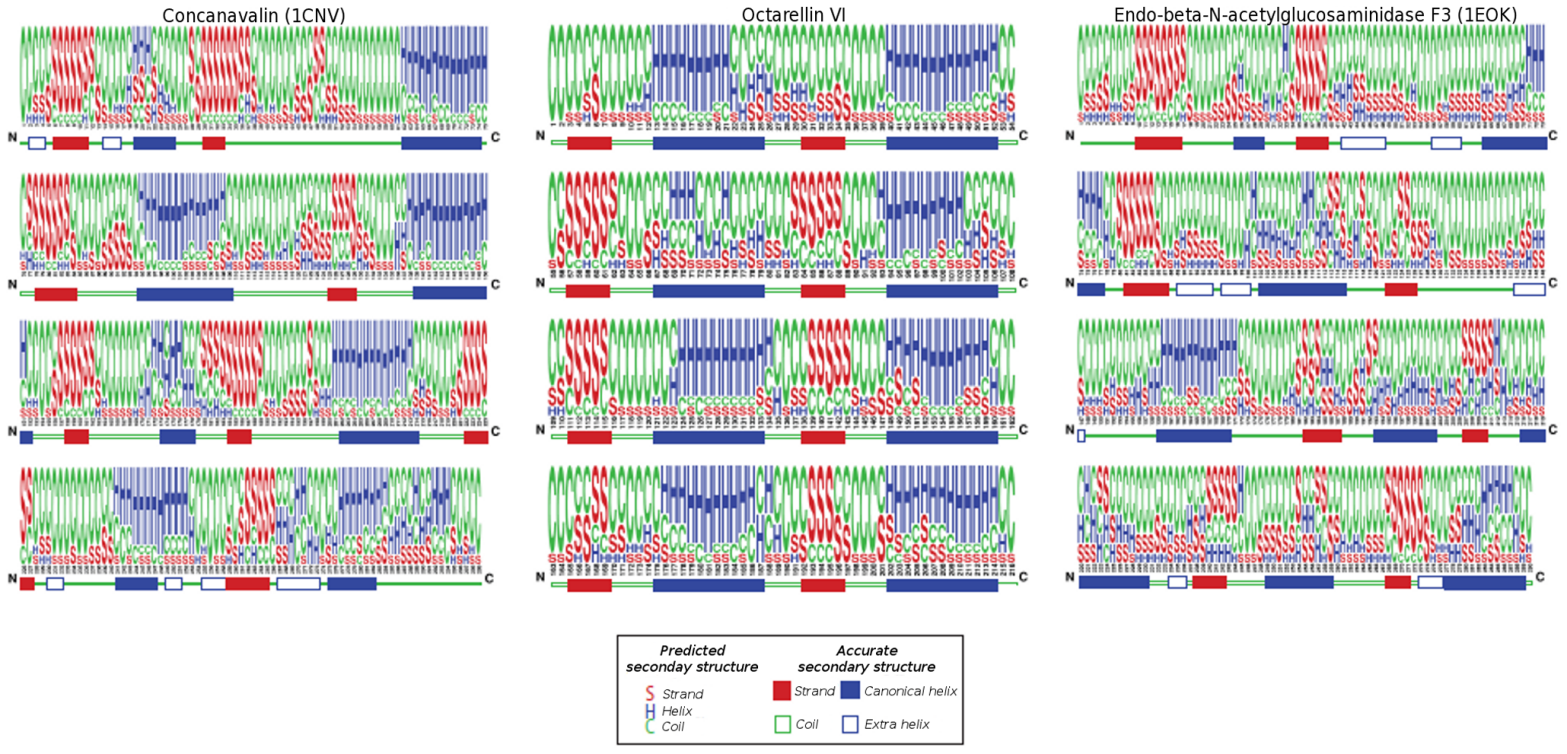
Testing the validity of predicted overall secondary structures. On the left and right, the validity of secondary structures predicted by JUFO is demonstrated for two proteins extracted from our control set (PDB ID 1 cnv and 1 eok). The upper line represents the predicted propensity for each secondary structure type at each position (coil, helix, or strand), while the lower line represents the actual secondary structure elements (based on the 3D crystal structure) listed in the corresponding PDB file. In the middle, the JUFO prediction for Octarellin VI is shown (upper line), as compared to the structure elements based on the model coordinates.

The average Rosetta energy per residue of the designed protein (the result of Rosetta’s energy function), −2.45 Rosetta energy units per residue, falls within the range of per residue energies observed for a set of 18 crystal structures of TIM barrels (−2.29±0.18 Rosetta energy units per residue, see Table 1). A secondary structure prediction by JUFO [48] identified 7 α-helices and 5 β-strands in the protein, the remaining α-helix and three β-strands being identified at a reduced confidence level (Fig. 4). The overall secondary structure prediction accuracy was comparable to that of predictions performed on a set of 18 natural TIM-barrel crystal structures (−0.62 vs. −0.55±0.12). We further performed a fold recognition analysis of the Octarellin VI sequence, checking the ability of our designed sequence to fold into a TIM-barrel, even though a Blast analysis revealed no similarity between Octarellin VI and any known protein (data not shown). The webservers I-Tasser, PsiPred, and 3D-Jury were used for this analysis. As best template, these webservers identified respectively bacterial luciferase (PDB code 1LUC), dihydrodipicolinate synthase (PDB code 2PUR), and 3D-Jury identified 2-keto-3-deoxygluconate aldolase (PDB code 1W37). All three of these proteins have a TIM-barrel fold.

**Figure 5:**
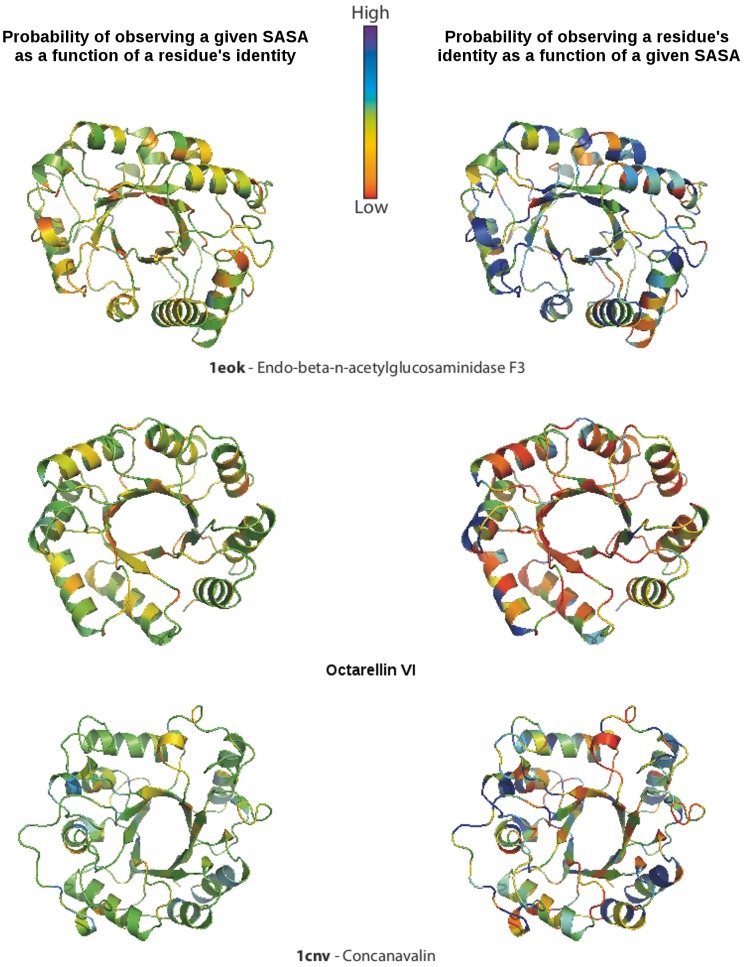
Residue packing in Octarellin VI as assessed by SASA analysis. SASA probabilities are shown for each residue of Octarellin VI and of two natural (β/α)_8_ barrels.

The quality of residue packing was assessed by SASA analysis. On the basis of the overall SASA scores, Octarellin VI appears less tightly packed than the 18 crystal structures (3.80 vs. an average of 1.49±1.09). The comparison appears more favorable, however, when one looks at the overall probability of observing the predicted exposure for a specific amino acid (0.34 vs. 0.46±0.05). Figure 5 shows, residue by residue, the probability of observing the predicted SASA for the amino acid present at each position and the probability of observing the expected residue given the SASA value determined at that position. From these figures, one can see that the solvent accessibility of the designed structure falls within acceptable limits.

In terms of amino acid categories, the amino acid composition of the synthetic TIM is comparable to that of natural (β/α)_8_ barrel proteins (see Table 2), but two categories stand out: first, the percentage of small amino acids is higher than expected (31.0% vs. 21.9% ±4.6%); this is likely mainly due to the fact that the glycine content of the designed protein is higher than the average content observed in our control set (18.1% vs. 7.9% ±1.7%). Second, the aromatic content of the designed protein is higher than expected (20.4% vs. 11.8% ±2.4%) because of our filter forcing the inclusion of aromatics in the designed sequences and our decision to include only nonpolar amino acids in the core.

**Figure 6:**
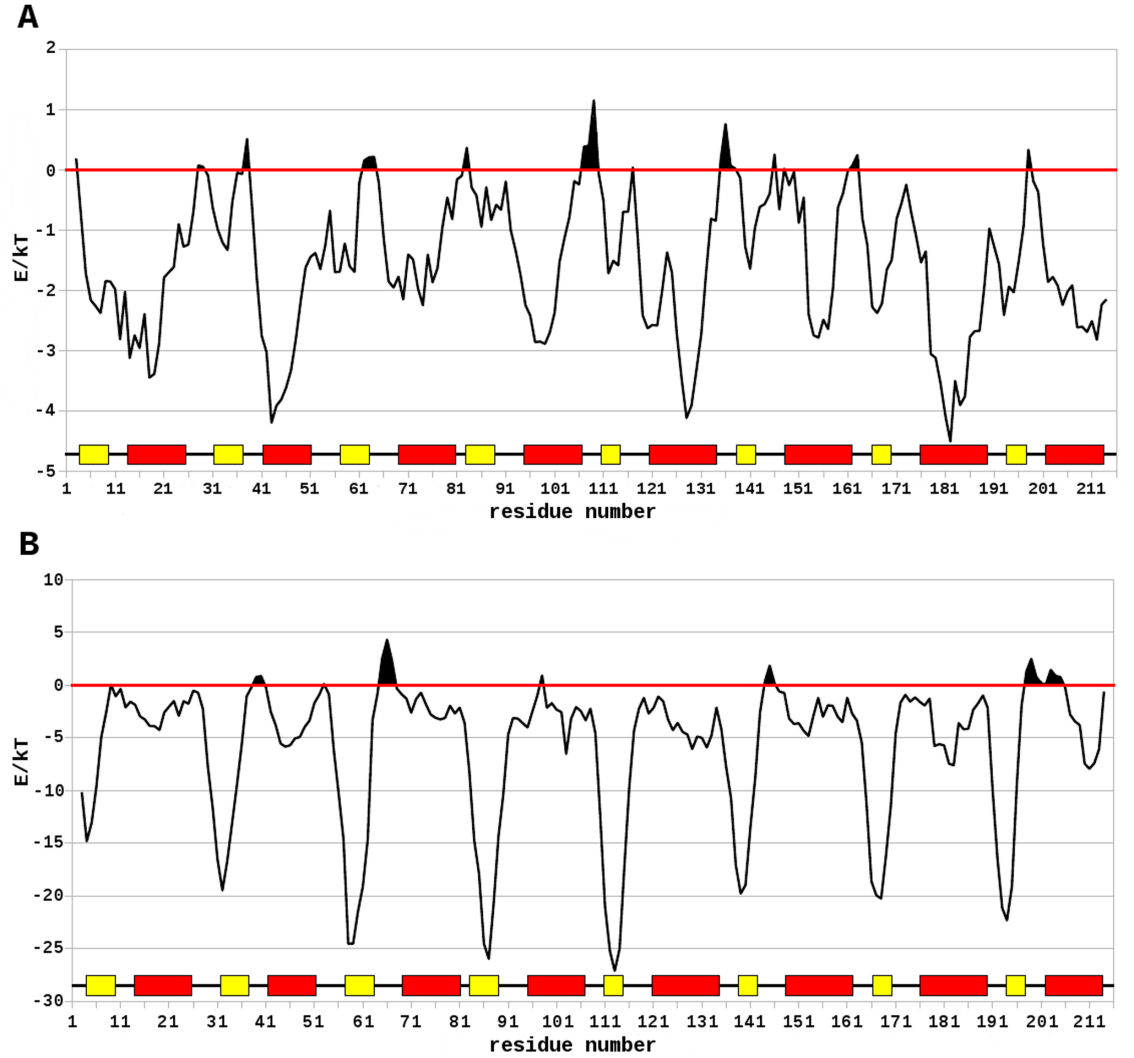
Local energy analysis by Anolea and ProsaII. (A) ProsaII analysis reveals that most of the protein has low energy, with the exception of some loop regions. (B) Anolea also shows high energy only in the loop regions.

The designed protein shows good stereochemical features. The Ramachandran plot revealed only 4 residues (1.9%) in a non-allowed region (data not shown): residues Arg 3, Ala 11, Ala 81, and Ala 144, all four present in loop regions. Local energy analyses with the Anolea (Fig. 6B) and ProsaII (Fig. 6A) webservers revealed similar high percentages of residues in the structure having a favorable low energy (92% observed with Anolea). Interestingly, helices showed the lowest local energy levels in the ProsaII analysis, as opposed to strands in the Anolea analysis. In both cases, however, the loop regions showed the highest local energy levels.

### Molecular Dynamics Simulations show the Structural Stability of the Designed Protein

**Figure 7:**
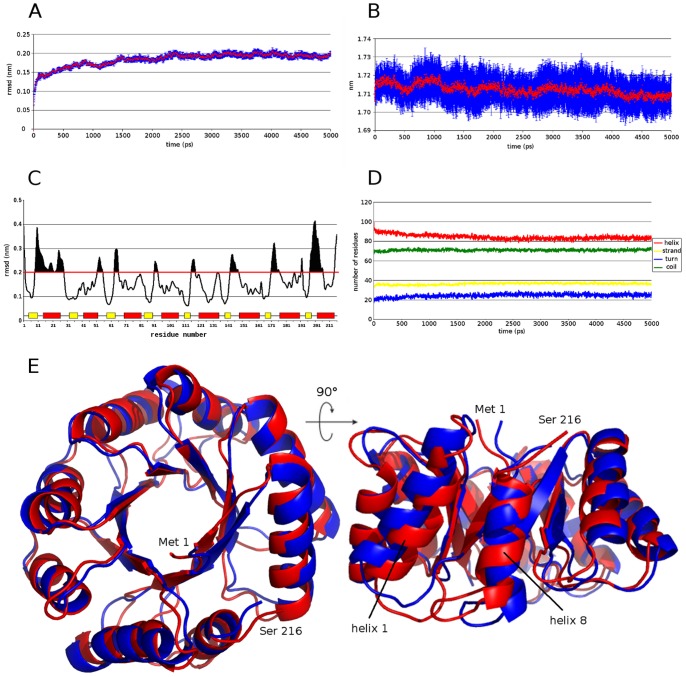
Molecular dynamics simulation analysis of Octarellin VI. (A) Root mean square deviation (rmsd) of the backbone through a 5-ns simulation. (B) Variation of the radius of gyration of Octarellin VI in the MD simulation. (C) Rmsd of each amino acid in the MD simulation. Yellow boxes represent strands and red boxes, helices. A threshold of 2 Å was defined, all rmsd values above this threshold being considered as real movements. The above-threshold area under the curve is represented in black in the latter situation. (D) Evolution of secondary structure contents in the course of the MD simulation. With DSSP software, the secondary structure content was calculated at each frame of the simulation and plotted as number of residues present for each secondary structure elements through the time of simulation. (E) Superposition between the original model of Octarellin VI (red) and the final model after MD simulation (blue). All secondary structure elements are maintained, with movements in loop zones and a rearrangement in helix 1 and 8 without loss of its secondary structure.

Despite its differences in amino acid content as compared to the control group, the Octarellin VI model showed good structural stability in MD simulations (Fig. 7). Ten different MD simulations were performed and the trajectories analyzed. The rmsd of the backbone reached a plateau at 3 ns, indicating no further change in the global structure, and an equilibrated structure (Fig. 7A). The radius of gyration remained constant throughout the simulations, in keeping with the stability suggested by the rmsd of the backbone. The secondary structure content also is proved to be stable: the helix content first decreased slightly, but remained stable after 2.5 ns of simulation. To test local displacements in the structure, a threshold of 2 Å was defined for the rmsd of each residue. According to this criterion, most of the movements in the protein were observed in the loop regions connecting strands with helices (Fig. 7C). Helix one and part of helix eight also showed displacements, but without any loss of structure. All these results suggest that our artificial protein is at a minimum global energy.

### Dynamic Light Scattering Indicates a Unique Population with a Hydrodynamic Diameter Close to that Expected for the Designed Protein

**Figure 8:**
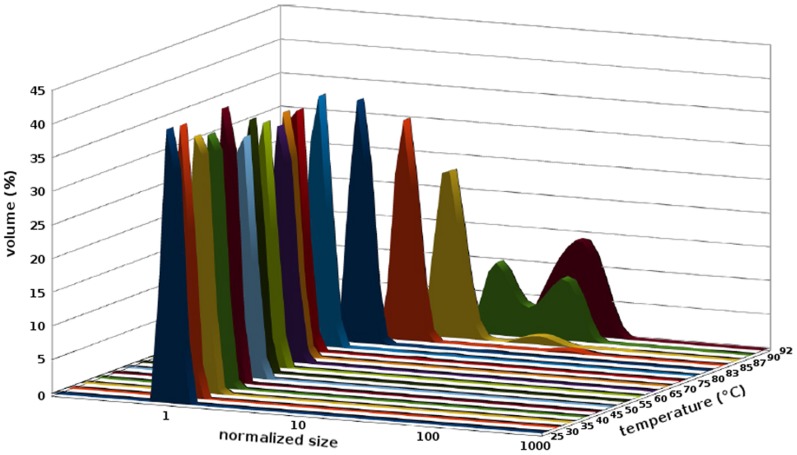
Effect of temperature on the particle size distribution. Dynamic light scattering was used to estimate the hydrodynamic diameter of Octarellin VI (at 5 µM concentration) at different temperatures. The average hydrodynamic diameter at 25 °C is 4.82 nm. Values are normalized and plotted on a logarithmic scale. Each temperature is represented by a different color.

To validate our model experimentally, the gene encoding Octarellin VI was expressed in *E. coli* BL21(DE3) as described under “Materials and Methods”. As the protein turned out to be completely insoluble in the bacteria, it was necessary to purify it from inclusion bodies and then to refold it. All measurements in this work were done on the refolded protein. We first performed a DLS analysis to measure fluctuations in particle size (hydrodynamic diameter) as a function of temperature in an interval ranging from 25°C to 92°C (Fig. 8)… At temperatures below 73°C, the average hydrodynamic diameter of the particles was found to be fairly constant (4.82±0.21 nm). The molecular weight of the protein, as estimated from these measurements, was 25.3 kDa. This is in excellent agreement with the theoretical molecular weight of 25.5 kDa and further indicates that Octarellin VI is a monomeric protein. Above 74°C, the particle size was found to increase significantly, from approximately 6 nm to more than 200 nm, and the size distribution profile of the protein population was found to shift from a very narrow single peak to several broader peaks (Fig. 8). These results suggest that heating above 74°C causes the protein to aggregate.

### Circular Dichroism Reveals a Folded Protein

**Figure 9:**
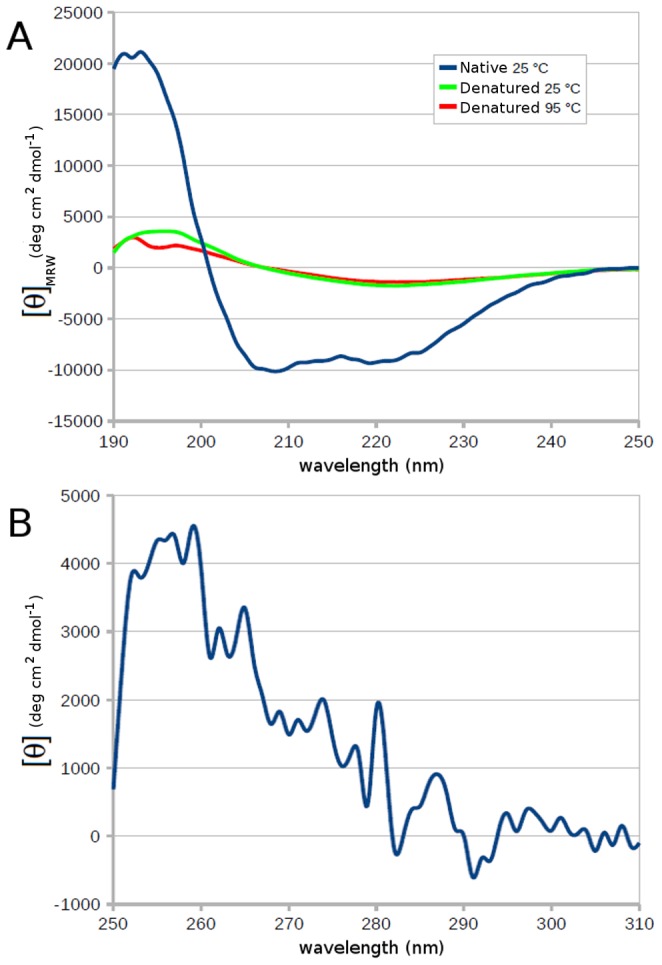
Circular dichroism spectra of the artificial protein. (A) Far-UV spectrum of Octarellin VI recorded at 25°C, after denaturation at 95°C, and after cooling from 95 to 25°C. (B) Near-UV spectrum of Octarellin VI at 25°C.

To check whether the refolded Octarellin VI adopts the predicted secondary structure, we measured CD spectra in the far UV. Two minima were observed close to 222 nm and 208 nm, and the overall spectrum looked typical of that expected of an α/β protein (Fig. 9A). A secondary structure analysis was performed with DichroWeb to estimate the percentage of each type of secondary structure. The spectrum of the protein refolded in the presence of NV10 gave good quality data down to 190 nm and hence, the secondary structure content of the protein was calculated and is given in Table 3. Data are in good agreement with those obtained for *T. Maritima* TIM (PDB code 1B9B), a natural thermostable α/β-barrel protein with 250 amino acids. Furthermore the analysis performed using the Dichroweb server indicated an average content of 3.8 helices per 100 residues, yielding a total value of about 8.2 helix segments in the protein, which is in complete agreement with our 8-helix design.

CD spectra were also obtained in the near-UV region. Absorption bands were observed, indicating that a number of aromatic side chains are held in a rigid environment. This suggests the presence of a tertiary structure (Fig. 9B).

### Thermal and Chemical Denaturations Monitored by Circular Dichroism and Tryptophan Fluorescence Reveal an Unfolding Transition

To test the stability of the protein and observe its unfolding, thermal and chemical denaturations were performed.

**Figure 10:**
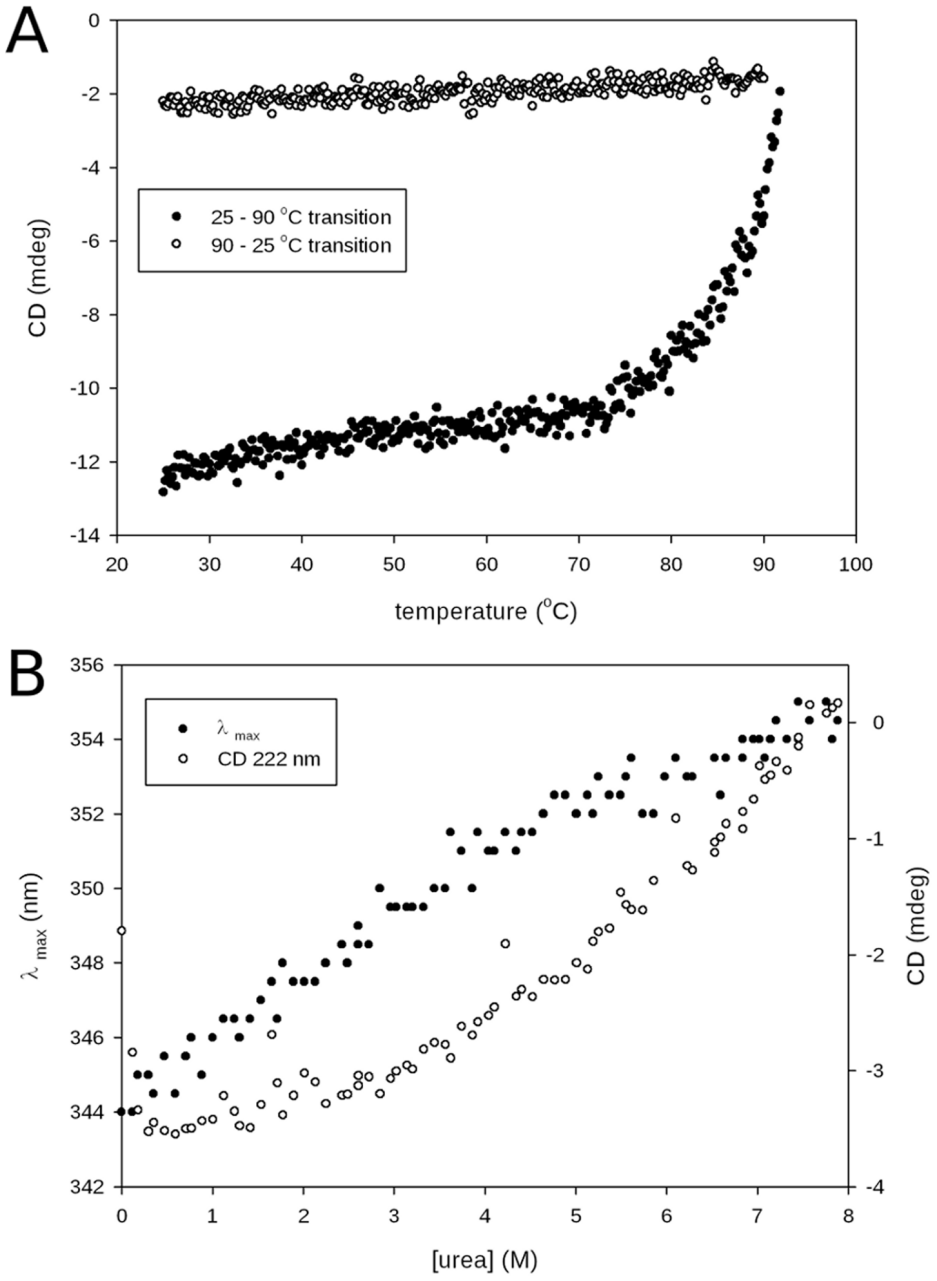
Urea- and heat-induced unfolding of the artificial protein. (A) Heat-induced unfolding monitored by measuring the CD signal at 222 nm; after unfolding, the CD signal at 222 nm was monitored during cooling from 95 to 25°C. (B) Change in the λ_max_ of fluorescence emission (after excitation at 280 nm) and change in the CD signal at 222 nm as a function of the urea concentration. The change in λ_max_ of fluorescene emission shows a shift from 344 nm (folded) to 355 nm (unfolded) as the urea concentration increases, and the same behavior is observed for the CD signal at 222 nm.

Heat denaturation was monitored by CD in the far-UV region (at 222 nm). The protein appeared stable up to 70°C (Fig. 10A, shift from 25 to 92°C), but above this temperature, heat-induced unfolding occurred, and this process was irreversible (Fig. 10A, shift from 92°C to 25°C). This result is in agreement with the DLS data (Fig. 8) showing that Octarellin VI remains stable and maintains its secondary structure content even at 70°C. Together, the DLS and CD data suggest that when the protein starts to unfold, stable aggregates appear (Fig. 8 and Fig. 10A).

Chemical denaturation of Octarellin VI (with urea) was monitored by recording tryptophan fluorescence and the CD signal at 222 nm. Increasing the urea concentration caused the wavelength of the emission maximum to shift from 344 nm to 355 nm. This is typical of the transition from a folded protein, where the tryptophans are protected in the core, to an unfolded protein, where the tryptophans are fully exposed to the solvent. The CD signal at 222 nm, which revealed the stability of the protein’s secondary structure, also showed a continuous decrease in the signal with the same profile as for the fluorescence assay.

Both techniques (Fig. 10A and 10B) showed a monotonous signal change upon unfolding, instead of a typical sigmoid profile. This suggests a noncooperative transition.

## Discussion

### Pushing the Size Limit

With the 100-residue protein Top7 [17], Rosetta is the only protein design protocol demonstrated to have yielded *de novo*, without the help of a scaffold protein, a model close to reality. In the wake of this and other successes, we have used Rosetta to design a protein twice as long, intended to adopt the (β/α)_8_ fold. We have thus designed, produced in *E. coli*, and purified the 216-residue protein Octarellin VI.

Our computational analyses of Octarellin VI suggest favorable overall structural energetics and highlight a resemblance to natural (β/α)_8_ barrel proteins as regards amino acid composition (apart from an overabundance of glycine and aromatic residues), predicted energetics, and predicted secondary structure features. Our experimental data are also encouraging: purified Octarellin VI shows a stable tertiary structure with the expected α-helix and β-sheet contents (as suggested by our CD and tryptophan fluorescence data) and high resistance to heat-induced unfolding.

In comparison with our previous work [7], Octarellin VI does not appear to show a big improvement, because it displays the same negative feature, the insolubility. However, the protocol implemented in Rosetta considers all the amino acids, while the proline residues were not allowed in the Octarellin V design. This new protein shows a better thermo stability, with an apparent Tm of 85 °C vs 65 °C for Octarellin V. Also, *in silico* simulation to test protein stability (Figure 7) shows a correct relationship between primary and tertiary structure in the Octarellin VI model. The same simulation for Octarellin V model shows more movements and changes in the global position of its atoms, leading at the end of the simulation to a structure where the rmsd with the original model is more than 5 Å (data not shown) while maintaining a (β/α)_8_ structure. This data indicates that the new protocol implemented into Rosetta enables to create a protein model where the primary structure has a better relationship with the tertiary structure.

Yet the protein is not soluble enough to allow determining its 3D structure by X-ray analysis, and shows apparently noncooperative unfolding.

### Solubility

Historically, attempts to design artificial TIM barrels *de novo* have often produced proteins with low solubility. In the present case, we think this problem is at least partially linked to the design methodology, which seems to produce excessively hydrophobic patches on the protein surface. The observed excess of glycine and aromatic residues might contribute to the problem [59], [60] by causing hydrophobic patches to appear, decreasing the proportion of polar residues at the protein surface, and favoring stabilization of intermediates liable to aggregate during the folding process. While the restriction to only polar amino acids to the surface could be a solution to avoid the appearance of hydrophobic patches, this approximation is far from the reality of a natural protein, where some hydrophobic amino acids in the surface are required to stabilize its structure [61].

Moreover, because glycine lacks a side chain, glycine residues increase the conformational space (or perhaps the dynamics, flexibility) of the unfolded polypeptide chain, rendering the unfolded state entropically favorable. This results in stabilization of the unfolded state and hence in a global reduction of the free energy of unfolding [62], [63].

The high glycine content is an artefact of the design process. Initially, the loop residues were set as glycines, to be ‘mutated’ by Rosetta in successive design rounds. For this, Rosetta can search a database of 6-residue loops contained in the PDB. Despite this feature, the initial glycines were not readily removed.

There are several instances where Rosetta users have had to make manual adjustments. The designers of Top7, for instance, had to restrict the protein’s twenty-two surface β-sheet positions to polar amino acids [17], and in the recent *de novo* design of a molecular switch, Ambroggio and Kuhlman found it necessary to constrain exploration of the sequence space by using an energy function derived from multisequence alignments of well-conserved members of their design target superfamily [18], [64]. We believe that these manual modifications have been necessary because the energy terms in the Rosetta potential only provide an accurate description of solvation effects, without explicitly discouraging aggregation. Yet protein aggregation is a phenomenon that goes beyond solvation, as it includes not just the energetics of the interaction with the solvent but also nonspecific interactions of the protein with itself. A newer version of the Rosetta potential might help to overcome this limitation [65]. Experimentally, furthermore, the choice of buffer can greatly influence the solubility of a designed protein. Understanding such effects might help to improve the design process.

### Folding/Unfolding

The relative roughness of the folding free energy landscapes of several (β/α)_8_ barrel superfamilies has been widely explored [6], [7], [66], [67], [68], [69], [70], [71], [72], [73], [74], [75]. At first glance the TIM-barrel topology appears as a monodomain structure, but many biophysical measurements have highlighted discrepancies between the very complex folding pathways observed and this simple picture. Actually, (β/α)_8_ barrels tend to behave more like multidomain proteins, with sequential folding and unfolding of subdomain folding units [67], [76]. Explaining these hierarchical folding patterns [74], [77] requires partitioning the unfolded state between off-pathway transient intermediate species with substantial secondary structure and stability [78] and on-pathway equilibrium intermediate species [79].

Our experimental results suggest that while we have succeeded in creating a thermodynamically stable protein, its folding kinetics might differ considerably from that of natural small proteins and might involve multiple pathways and intermediate-state populations. Rosetta optimizes only for thermodynamic stability, without taking pathways and folding kinetics into account. The apparent noncooperative unfolding of Octarellin VI might be due to this fact. With a protein of more than 200 amino acids, the conformational space is larger than with a 100-amino-acid protein, and not taking into count the folding pathway might contribute to the problem. At this point, it is necessary to mention that we performed a 2D-NMR characterization over our artificial protein (data not shown). The result is not what we were expecting, as it shows that our protein is indeed not well folded under the tested experimental conditions. We believe this issue could be due to a wrong folding arising from the renaturation protocol. Clearly the possibility to get a soluble protein will allow a better characterization of the protein. Changing the expression system to yeast or cell lines like HEK cells could be a way to produce soluble proteins. Indeed, while the primary structure of a protein defines its tertiary structure, the environment (*in vivo* or *in vitro*) has a clear influence and impact on the final structure [2].

### What Next?

Future attempts to design large proteins will thus need to integrate an adequate amino acid environment potential encompassing both solvation and aggregation energetics. Ideally, they should also incorporate some assessment of potential folding pathways and of the folding kinetics of the designed proteins. This will require learning more about sequence-structure relationships and protein folding pathways. Secondary structure predictions in combination with local energy evaluations might be a good starting point at the present time, but it remains a challenge to perform *de novo* folding simulations with trajectories approaching those observed in nature with a sufficient level of accuracy, and to use this information in the design process. Furthermore, on the basis of proteins such as Octarellin VI, one should be able to create, by directed evolution, variants that are more soluble and whose structure can be determined accurately. With a database of such mutants and their characteristics, it might be possible to deduce rules or parameter changes that could be introduced into protocols such as Rosetta.

### Conclusions

We have used the Rosetta computational protein design protocol to design Octarellin VI, a 216-residue artificial protein modeled on the (β/α)_8_ barrel fold. The protein shows evidence of tertiary structure and high resistance to heat-induced unfolding, but low solubility and apparently noncooperative unfolding in the presence of urea. Our results highlight the need to incorporate into design protocols some assessment of potential folding pathways and of the folding kinetics of the designed proteins. Such methods remain to be developed. Secondary structure predictions, *de novo* folding simulations, and directed evolution could be starting points.
